# Data-driven, two-stage machine learning algorithm-based prediction scheme for assessing 1-year and 3-year mortality risk in chronic hemodialysis patients

**DOI:** 10.1038/s41598-023-48905-9

**Published:** 2023-12-05

**Authors:** Wen-Teng Lee, Yu-Wei Fang, Wei-Shan Chang, Kai-Yuan Hsiao, Ben-Chang Shia, Mingchih Chen, Ming-Hsien Tsai

**Affiliations:** 1 Division of Nephrology, Department of Internal Medicine, Shin-Kong Wu Ho-Su Memorial Hospital, No. 95, Wen-Chang Rd, Shih-Lin Dist., Taipei, 11101 Taiwan; 2 Department of Medicine, Fu Jen Catholic University, No. 510, Zhongzhen Rd., Xinzhuang Dist., New Taipei City, 24205 Taiwan; 3 Artificial Intelligence Development Center, Fu Jen Catholic University, No. 510, Zhongzhen Rd., Xinzhuang Dist., New Taipei City, 24205 Taiwan; 4 Graduate Institute of Business Administration, College of Management, Fu Jen Catholic University, No. 510, Zhongzhen Rd., Xinzhuang Dist, New Taipei City, 24205 Taiwan

**Keywords:** Nephrology, Kidney, Kidney diseases, Renal replacement therapy, Mathematics and computing, Computational science, Computer science

## Abstract

Life expectancy is likely to be substantially reduced in patients undergoing chronic hemodialysis (CHD). However, machine learning (ML) may predict the risk factors of mortality in patients with CHD by analyzing the serum laboratory data from regular dialysis routine. This study aimed to establish the mortality prediction model of CHD patients by adopting two-stage ML algorithm-based prediction scheme, combined with importance of risk factors identified by different ML methods. This is a retrospective, observational cohort study. We included 800 patients undergoing CHD between December 2006 and December 2012 in Shin-Kong Wu Ho-Su Memorial Hospital. This study analyzed laboratory data including 44 indicators. We used five ML methods, namely, logistic regression (LGR), decision tree (DT), random forest (RF), gradient boosting (GB), and eXtreme gradient boosting (XGB), to develop a two-stage ML algorithm-based prediction scheme and evaluate the important factors that predict CHD mortality. LGR served as a bench method. Regarding the validation and testing datasets from 1- and 3-year mortality prediction model, the RF had better accuracy and area-under-curve results among the five different ML methods. The stepwise RF model, which incorporates the most important factors of CHD mortality risk based on the average rank from DT, RF, GB, and XGB, exhibited superior predictive performance compared to LGR in predicting mortality among CHD patients over both 1-year and 3-year periods. We had developed a two-stage ML algorithm-based prediction scheme by implementing the stepwise RF that demonstrated satisfactory performance in predicting mortality in patients with CHD over 1- and 3-year periods. The findings of this study can offer valuable information to nephrologists, enhancing patient-centered decision-making and increasing awareness about risky laboratory data, particularly for patients with a high short-term mortality risk.

## Introduction

Patients on hemodialysis (HD) had a significantly higher mortality rate than the general population^1–4^. The life expectancy of patients with chronic hemodialysis (CHD) can be affected by underlying conditions such as aging, anemia, C-reactive protein, hypoalbuminemia, phosphorus, previous cardiovascular event, and dialysis adequacy^2,5–10^. Temporary vascular catheter could also be an independent risk factor of mortality in patients with CHD^6,11^. Previous studies already reported some clinical factors associated with mortality risks in patients with CHD; however, patients with end-stage kidney disease present considerable heterogeneity in the disease pattern with broad comorbidities^12,13^. Thus, making a survival outcome prediction model via limited clinical indicators remains challenging.

Various approaches have been attempted to modify the mortality prediction models for patients with CHD. The systematic review of Panupong Hansrivijit et al. demonstrated the precision of factors in predicting mortality in patients with chronic kidney disease, including those undergoing HD and peritoneal dialysis^14^. Moreover, Chava L. Ramspek et al. conducted a systemic review and further meta-analysis of independent external validation studies to determine the most ideal predictive performance study^13^. Mikko Haapio et al. performed two developed prognostic models of newly entered mortality prediction for patients with chronic dialysis via logistic regression (LGR) with stepwise variable selection and showed some variables to establish a practical, fine-performing model; however, they may overestimate the mortality risk because of considerably the lower mortality rate observed in the newer cohort^10^.

Recently, artificial intelligence (AI) has become increasingly popular in the field of patient survival/mortality analysis. Patients with CHD can provide robust serum laboratory data during HD treatments, considering that physicians commonly use them to monitor patients' condition. Machine learning (ML), a branch of AI that imitates human intelligence by incorporating and analyzing available data, is widely utilized in this context^15–17^. It has a unique potential for predicting survival outcomes and identifying mortality risk factors in patients with CHD. Powerful prediction models for patients with CHD have been developed using ML methods such as LGR, random forest (RF), and eXtreme gradient boosting (XGB). The research field encompasses diverse areas, including dialysis adequacy predictions^18^, survival prognostic prediction^19–21^, and time-dependent adverse event prediction^22^.

Identifying mortality risk factors in patients with CHD may facilitate early intervention and improve outcomes. Given that various ML techniques are still undergoing development and competition^23^, relying on a single approach may not consistently outperform others in all conditions. Therefore, the performance and accuracy of these techniques should be evaluated comprehensively. Hence, this study aimed to investigate the importance of risk factors identified using multiple ML methods. We also sought to establish a two-stage ML algorithm-based prediction scheme by comparing the accuracy and consistency of different ML methods to determine the most suitable model and identify common risk factors among patients with CHD who experienced mortality events in different years.

## Methods

### Study design and population

This retrospective observational cohort included 805 patients who received HD at Shin-Kong Wu Ho-Su Memorial Hospital between December 2006 and December 2012. The primary objective of creating this cohort was to assess the impact of reducing intra-dialysis phosphorus on mortality in patients with CHD was evaluated^24^. This cohort excluded the following criteria: (1) a history of hospitalization for acute events, including cardiovascular, cerebrovascular, and infectious diseases; (2) newly active diseases within 3 months before the data collection; and (3) missing information. After completing the essential data preprocessing steps before applying machine learning (ML) methods in the present study, a total of 800 patients were selected, as they had complete and relevant data required for further analysis. These patients were deemed suitable for inclusion in the study, ensuring a comprehensive dataset for subsequent ML modeling and analysis.

This study conformed to the principles of the Declaration of Helsinki, with approval by the Ethics Committee of the Shin-Kong Wu Ho-Su Memorial Hospital (protocol No.: 20220112R). Given that our study was based on medical records and data review, informed consent was relinquished by the Ethics Committee of the Shin-Kong Wu Ho-Su Memorial Hospital. Furthermore, patient information was anonymized and de-identified before the analysis.

### Data collection

This study included 44 variables, such as demographic, biochemical laboratory data, and underlying comorbidities with disease and drugs (e.g., diabetic mellitus, hypertension, cardiovascular disease (CVD), chronic obstructive pulmonary disease, renin–angiotensin–aldosterone system blocker, antiplatelet drug, statin, and beta-blocker). We incorporated all these factors into our analysis because of their critical relevance and strong association to clinical outcomes in CHD patients. Additionally, these parameters were easily obtainable in clinical practice.

In this study, CVD was characterized as a composite of various conditions significantly affecting the mortality of CHD patients. These encompass coronary artery diseases, heart failure, hypertensive heart disease, arrhythmias, valvular heart disease, peripheral artery disease, and thromboembolic disease. Within our clinical practice, routine follow-ups for CHD patients encompassed serum biochemical laboratory assessments, including dialysis quality, electrolyte levels, hemogram, nutritional status, iron profile, lipid profile, and parathyroid function.

The biochemical laboratory data in our study included urea kinetics (Kt/V), urea reduction ratio (URR), blood urea nitrogen (BUN, mg/dL) from pre- and post-HD, creatinine (Cr, mg/dL) from pre- and post-HD, sodium (Na, mEq/L) from pre- and post-HD, potassium (K, mEq/L) from pre- and post-HD, ionized calcium (iCa, mEq/L), phosphate from pre- and post-HD (P, mg/dL), intact parathyroid hormone (iPTH, pg/mL), alkaline phosphatase (IU/L), aspartate aminotransferase (AST, U/L), alanine transaminase (ALT, U/L), total bilirubin (mg/dL), aluminum (ng/mL), uric acid (mg/dL), albumin (g/dL), triglyceride (mg/dL), total cholesterol (mg/dL), high-density lipoprotein (mg/dL), low-density lipoprotein (LDL, mg/dL), hemoglobin (g/dL), hematocrit (%), mean corpuscular volume (MCV, fL), ferritin (μg/L), iron (μg/dL), total iron binding capacity (TIBC) (μg/dL), transferrin saturation (TSAT) (%), ante cibum (AC) blood glucose (mg/dL), post cibum (PC) blood glucose (mg/dL), total protein (g/dL), and cardiothoracic ratio (CTR) (%). Following an 8-h fast for routine biochemical testing, blood samples were collected from patients both before and after their dialysis session. Without using a tourniquet, samples were collected from tunneled catheters, arteriovenous fistulas, or grafts; the initial sample was discarded from heparin-primed catheters.

### Statistical analyses

Continuous data were reported as mean ± standard deviation and categorical data were expressed as numbers (%) of patients. To compare the means of continuous variables, analysis of variance (ANOVA) was employed. Additionally, the chi-square test (χ^2^ test) was utilized to compare categorical variables between different groups.

**Figure 1:**
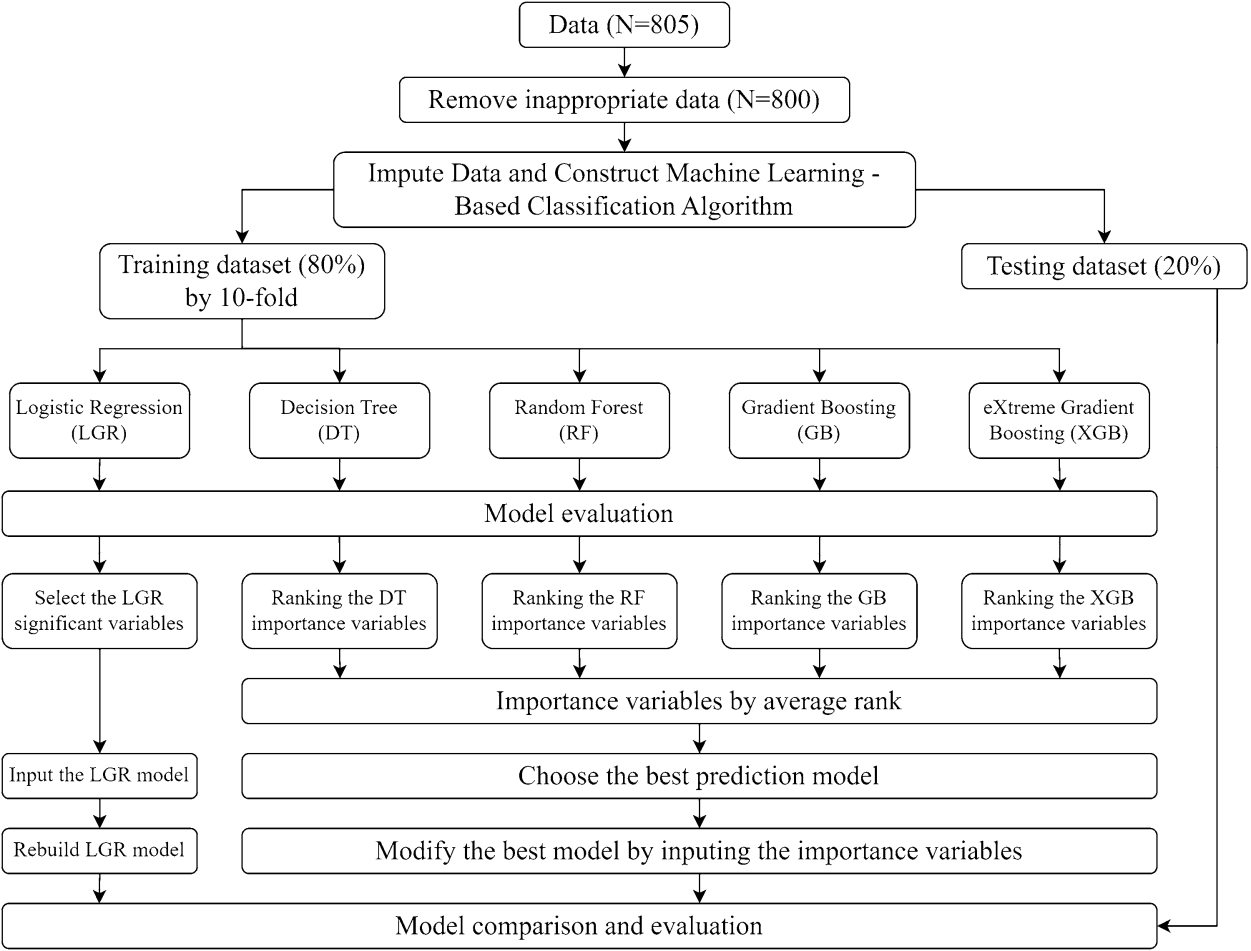
The algorithm of data-driven machine-learning methods in chronic hemodialysis patients.

Figure 1 presents the algorithm of data-driven ML methods in CHD patients. We divided the prediction subgroups into 1- and 3-year mortality. Each subgroup utilized ML algorithms to construct prediction models and evaluate the important factors. To establish the fundamentals of model building and validation, we followed a multistage process. First, we divided the patients into validation and testing datasets. The validation dataset comprised 80% of patients with CHD, while the remaining 20% constituted the testing dataset. Next, we employed a tenfold method on the validation dataset. This method involved dividing the dataset into 10 equal parts or folds, ensuring data randomization. Once the folds were established, we proceeded to build five different ML models: LGR, decision tree (DT), RF, gradient boosting (GB), and XGB. Through this approach, the performance of each model can be thoroughly validated and evaluated according to the different folds from the validation dataset. All five ML models were evaluated their respective indicators, including accuracy, sensitivity, specificity, and area under the curve (AUC).

After the evaluation of these ML models, we compared the results with those of the LGR model and the best modified model from other four. To achieve this, we selected the most significant risk variables from the LGR model alone, while the four remaining models (DT, RF, GB, and XGB) ranked such variables by averaging their rankings. We used these top variables from LGR to modify the rebuilt logistic regression (rebuilt LGR) model and modify the best model among the four remaining models by incorporating the variables with the highest average rank into the most suitable model. Finally, we compared the results with the best remodified model and the rebuilt LGR model through validation and compared the results with the previous training dataset. A two-tailed *p* value of < 0.05 was considered statistically significant. All statistical data were analyzed using R for MacOS version 4.2.1.

## Results

### Study population characteristics

We included 800 patients receiving HD, with 718 of them surviving at the 1-year mark and 519 surviving at the 3-year mark. Table 1 summarizes the participants’ basic characteristics, comorbidities, and laboratory data. The overall mean age of the study population was 63.30 ± 13.26 years. In the subgroup with a one-year observation, the mean age of the survival group was 62.73 ± 13.20 years, whereas the mortality group had a mean age of 73.55 ± 11.94 years. For the with a 3-year observation, the survival group had a mean age of 60.92 ± 13.04 years, while the mortality group had a mean age of 71.24 ± 11.43 years.

**Table 1:** The baseline characteristics of study population.

Variables	Total (n = 800)	1-year observation		3-year observation	
		Alive (n = 718)	Dead (n = 42)	Alive (n = 519)	Dead (n = 147)
Demography					
Male	405 (50.63)	362 (50.42)	24 (57.14)	276 (53.18)	69 (46.94)
Age (years)	63.30 ± 13.26	62.73 ± 13.20 **	73.55 ± 11.94 **	60.92 ± 13.04 **	71.24 ± 11.43 **
Hemodialysis time (years)	4.23 ± 4.65	4.33 ± 4.71	4.34 ± 4.22	4.62 ± 4.79	4.45 ± 4.50
Comorbidities and medications					
Diabetic mellitus	342 (42.75)	305 (42.48)	18 (42.86)	206 (39.69)	71 (48.30)
Hypertension	335 (41.88)	302 (42.06) *	10 (23.81) *	211 (40.66)	56 (38.10)
Cardiovascular disease	225 (28.13)	200 (27.86)	15 (35.71)	132 (25.43) *	54 (36.73) *
COPD	54 (6.75)	51 (7.10)	2 (4.76)	39 (7.51)	8 (5.44)
RAAS blocker	277 (34.63)	252 (35.10) *	6 (14.29) *	188 (36.22) *	33 (22.45) *
Anti-platelet	285 (35.63)	256 (35.65)	15 (35.71)	171 (32.95) *	62 (42.18) *
Statin	149 (18.63)	134 (18.66)	8 (19.05)	100 (19.27)	26 (17.69)
Beta blocker	159 (19.88)	144 (20.06)	7 (16.67)	114 (21.97)	22 (14.97)
Biochemical laboratory data					
Kt/V	1.34 ± 0.24	1.34 ± 0.22 **	1.49 ± 0.44 **	1.35 ± 0.22	1.37 ± 0.30
URR	0.73 ± 0.06	0.73 ± 0.06	0.76 ± 0.07	0.73 ± 0.06	0.74 ± 0.06
BUN (pre-HD) (mg/dL)	69.02 ± 18.27	69.31 ± 18.19 *	62.36 ± 20.71 *	69.41 ± 17.96	66.14 ± 19.96
BUN (post-HD) (mg/dL)	18.58 ± 7.33	18.75 ± 7.37 *	15.17 ± 7.32 *	18.54 ± 7.42	17.32 ± 6.62
Creatinine (pre-HD) (mg/dL)	9.41 ± 2.32	9.48 ± 2.28 **	8.03 ± 2.65 **	9.68 ± 2.28 **	8.39 ± 2.21 **
Creatinine (post-HD) (mg/dL)	3.09 ± 0.95	3.12 ± 0.93 **	2.57 ± 1.19 **	3.15 ± 0.94 **	2.79 ± 0.93 **
AC blood sugar (mg/dL)	114.16 ± 57.07	113.85 ± 56.65	116.94 ± 64.04	110.73 ± 55.20 *	123.28 ± 64.63 *
PC blood sugar (mg/dL)	220.67 ± 94.27	222.27 ± 94.50	228.10 ± 106.23	224.25 ± 98.24	232.10 ± 99.19
Sodium (Na, mEq)	139.28 ± 3.70	139.35 ± 3.54	138.95 ± 5.73	139.45 ± 3.44	139.18 ± 4.39
Potassium (pre-HD) (K, mEq)	4.70 ± 0.69	4.72 ± 0.69 *	4.40 ± 0.74 *	4.74 ± 0.67	4.57 ± 0.70
Potassium (post-HD) (K, mEq)	3.20 ± 0.37	3.20 ± 0.37	3.14 ± 0.43	3.19 ± 0.36	3.17 ± 0.35
Ionized calcium (iCa, mEq)	4.62 ± 0.45	4.62 ± 0.45	4.61 ± 0.43	4.61 ± 0.44	4.69 ± 0.46
Phosphate (pre-HD) (P, mg/dL)	5.20 ± 1.48	5.21 ± 1.48 *	4.61 ± 1.38 *	5.25 ± 1.48	4.98 ± 1.54
Phosphate (post-HD) (P, mg/dL)	2.21 ± 0.57	2.23 ± 0.57 **	1.88 ± 0.62 **	2.24 ± 0.58 *	2.07 ± 0.54 *
iPTH (pg/mL)	166.54 ± 191.61	169.86 ± 196.23 *	98.37 ± 95.58 *	174.30 ± 194.17 *	122.07 ± 133.60 *
Alkaline phosphatase (IU/L)	97.70 ± 86.00	96.14 ± 83.50 *	131.74 ± 135.32 *	96.00 ± 91.97	111.63 ± 86.03
AST (U/L)	22.89 ± 22.21	22.54 ± 22.80 *	30.38 ± 17.71 *	22.37 ± 25.46 *	27.09 ± 16.43 *
ALT (U/L)	23.51 ± 22.37	23.31 ± 22.81	27.45 ± 17.89	23.12 ± 23.67	26.90 ± 22.68
Total bilirubin (mg/dL)	0.39 ± 0.23	0.39 ± 0.20 **	0.54 ± 0.46 **	0.39 ± 0.21 *	0.44 ± 0.31 *
Total protein (g/dL)	7.33 ± 0.65	7.35 ± 0.64	7.37 ± 0.79	7.37 ± 0.63	7.42 ± 0.70
Albumin (g/dL)	4.14 ± 0.40	4.16 ± 0.38 **	3.76 ± 0.54 **	4.20 ± 0.38 **	3.98 ± 0.44 **
HDL (mg/dL)	50.57 ± 17.08	50.68 ± 17.11	48.06 ± 19.49	51.30 ± 17.31	50.33 ± 17.82
LDL (mg/dL)	104.56 ± 35.62	105.18 ± 34.52 *	92.17 ± 49.37 *	106.80 ± 35.12 *	98.52 ± 37.94 *
Total cholesterol (mg/dL)	174.73 ± 43.71	175.24 ± 42.86	161.42 ± 57.67	176.43 ± 42.05	169.22 ± 46.86
Triglyceride (mg/dL)	163.65 ± 148.97	165.35 ± 152.02	124.00 ± 87.27	160.10 ± 130.62	153.51 ± 110.25
Uric acid (mg/dL)	6.74 ± 2.27	6.75 ± 2.26	6.53 ± 2.01	6.81 ± 2.26 *	6.31 ± 2.41 *
Hemoglobin (g/dL)	10.31 ± 1.49	10.33 ± 1.41	10.53 ± 2.40	10.38 ± 1.45	10.48 ± 1.75
Hematocrit (%)	31.73 ± 4.31	31.78 ± 4.07	32.52 ± 6.97	31.95 ± 4.17	32.37 ± 5.05
MCV (fL)	93.30 ± 7.40	93.31 ± 7.26	93.94 ± 8.67	93.28 ± 7.44	93.82 ± 7.43
Iron (μg/dL)	75.01 ± 33.97	75.11 ± 33.72	73.08 ± 37.27	76.16 ± 34.37	73.13 ± 31.66
TIBC (μg/dL)	221.64 ± 46.53	222.06 ± 45.46	210.58 ± 62.15	221.92 ± 44.84	221.46 ± 51.00
TSAT (%)	34.54 ± 15.56	34.45 ± 15.29	36.28 ± 17.47	35.07 ± 15.86	33.61 ± 13.65
Ferritin (μg/L)	559.86 ± 363.00	566.61 ± 367.27	497.92 ± 292.65	574.45 ± 362.43	529.77 ± 279.26
Aluminum (ng/mL)	7.72 ± 7.66	7.48 ± 7.54 *	11.00 ± 8.84 *	7.36 ± 7.30 *	9.33 ± 9.30 *
CTR (%)	50.71 ± 6.82	50.50 ± 6.85 **	55.35 ± 5.96 **	49.96 ± 6.81 **	54.17 ± 6.34 **

*COPD* chronic obstructive pulmonary disease, *RAAS* renin–angiotensin–aldosterone system, *Kt/V* urea kinetic, *URR* urea reduction ratio, *BUN* blood urea nitrogen, *AC* Ante Cibum (before meals), *PC* Post Cibum (after meals), *iPTH* intact parathyroid hormone, *AST* aspartate aminotransferase, *ALT* alanine transaminase, *HDL* high-density lipoprotein, *LDL* low-density lipoprotein, *MCV* mean corpuscular volume, *TIBC* total iron binding capacity, *TSAT* transferrin saturation, *CTR* cardiothoracic ratio.

*p* values * * p value < 0.05 are and **p value < 0.001.

### Comparison of survival prediction performance among different ML methods

Table 2 shows the comparison of survival prediction performance among different ML models, including LGR, DT, RF, GB, and XGB, in terms of accuracy, sensitivity, specificity, and AUC. For the 1-year mortality prediction model, both RF and GB showed high accuracy and AUC in both validation and testing datasets. Specifically, RF had 0.948 accuracy in validation datasets and 0.941 in testing datasets, while GB had 0.949 and 0.941, respectively. Regarding AUC, RF obtained 0.734 in validation datasets and 0.806 in testing datasets, while GB had 0.737 and 0.793, respectively. For the 3-year mortality prediction model, RF had the highest accuracy in both validation and testing datasets (0.794 and 0.804, respectively), and its AUC values were 0.751 and 0.763, respectively, indicating solid performance. Overall, RF outperformed other models in terms of accuracy and AUC for all four study cutoff periods in both validation and testing datasets.

**Table 2:** Comparison of survival prediction performance among different machine learning models.

Methods	Validation dataset					Testing dataset				
	LGR	DT	RF	GB	XGB	LGR	DT	RF	GB	XGB
1-year mortality										
Accuracy	0.930	0.929	0.948	0.949	0.930	0.912	0.934	0.941	0.941	0.912
Sensitivity	0.161	0.043	0.020	0.063	0.161	0.044	0.156	0.000	0.022	0.044
Specificity	0.971	0.978	1.000	0.999	0.971	0.967	0.983	1.000	0.999	0.967
AUC	0.648	0.573	0.734	0.737	0.648	0.734	0.590	0.806	0.793	0.734
3-year mortality										
Accuracy	0.779	0.762	0.794	0.789	0.810	0.774	0.743	0.804	0.802	0.803
Sensitivity	0.343	0.297	0.075	0.154	0.182	0.243	0.286	0.096	0.171	0.175
Specificity	0.899	0.890	0.991	0.962	0.984	0.916	0.865	0.992	0.970	0.970
AUC	0.746	0.679	0.751	0.754	0.786	0.756	0.660	0.763	0.773	0.788

*LGR* logistic regression, *DT* decision tree, *RF* random forest, *GB* gradient boosting, *XGB* eXtreme gradient boosting, *AUC* area under curve.

### Ranking of CHD mortality risk variables using ML methods

**Figure 2:**
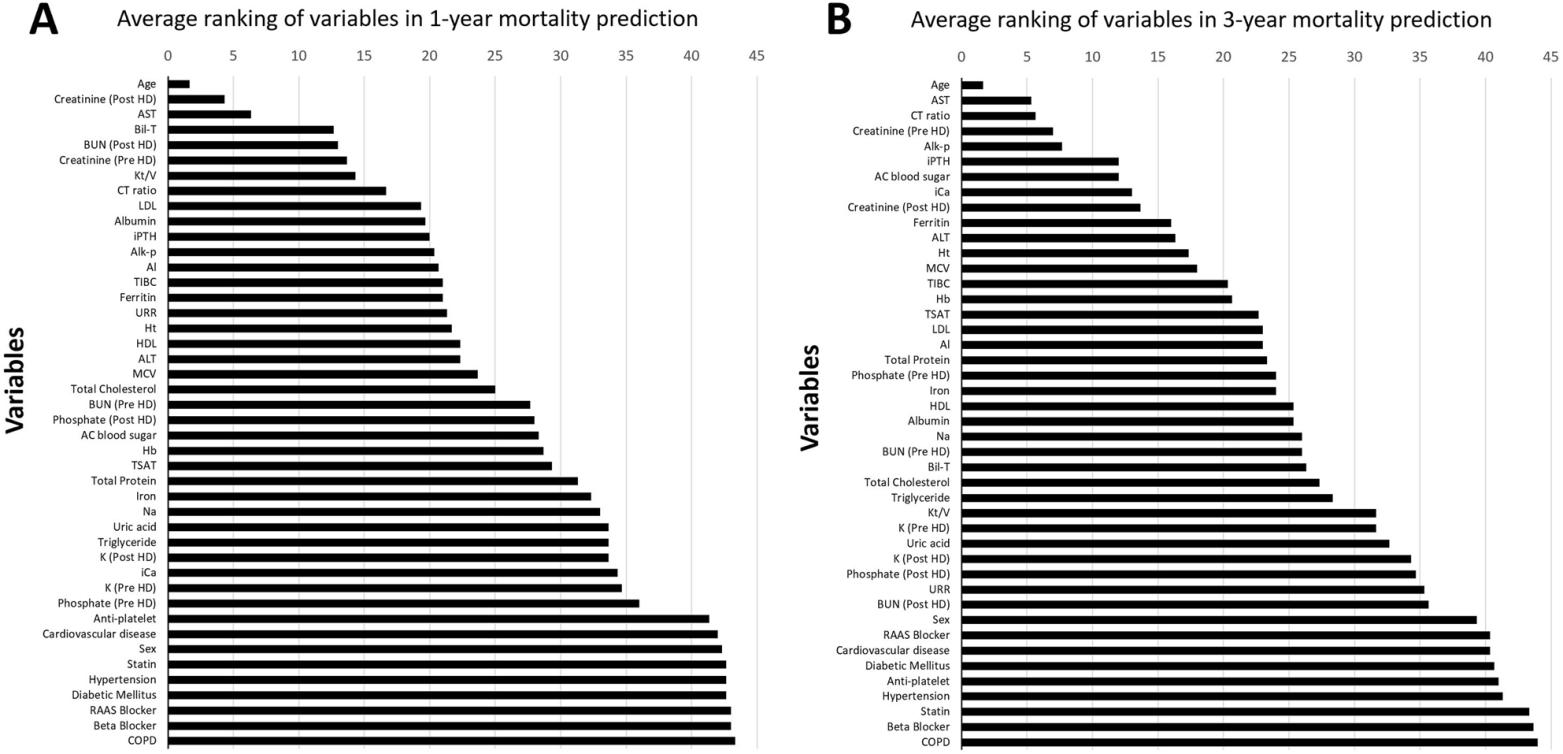
The average ranking of variables from four machine-learning models (decision tree, random forest, gradient boosting, and eXtreme gradient boosting ) in (**A**) 1-year and (**B**) 3-year mortality prediction. *COPD* chronic obstructive pulmonary disease, *RAAS* renin–angiotensin–aldosterone system, *Kt/V* urea kinetic, *URR* urea reduction ratio, *BUN* blood urea nitrogen, *AC* Ante Cibum (before meals), *PC* Post Cibum (after meals), *Na* sodium, *K* Potassium, *iCa* ionized calcium, *iPTH* intact parathyroid hormone, *Alk-p* alkaline phosphatase, *AST* aspartate aminotransferase, *ALT* alanine transaminase, *Bil-T* total bilirubin, *HDL* high-density lipoprotein, *LDL* low-density lipoprotein, *Hb* hemoglobin, *Ht* hematocrit, *MCV* mean corpuscular volume, *TIBC* total iron binding capacity, *TSAT* transferrin saturation, *Al* Aluminum (ng/mL), *CTR* cardiothoracic ratio.

**Figure 3:**
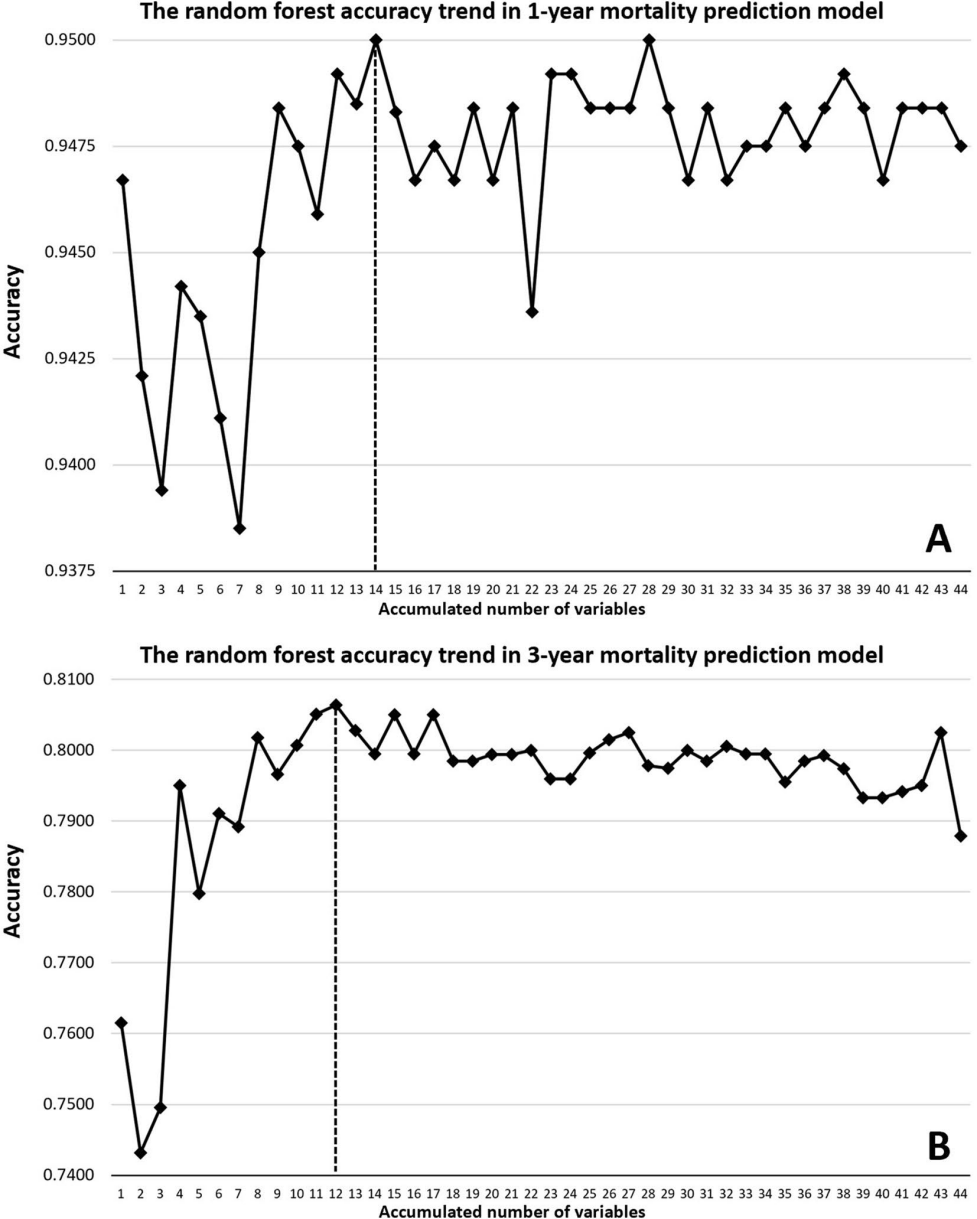
The random forest accuracy trend by the accumulated number of variables for (**A**) 1-year mortality and (**B**) 3-year mortality prediction models.

Figure 2 presents the average ranking of variables from four ML models (DT, RF, GB, and XGB) in 1- and 3-year mortality predication. Given that RF had the highest accuracy and AUC results in both the validation and testing datasets, we selected it as the optimal modified model for our study and subsequently put the top average ranking variables. Figure 3 illustrates the RF accuracy trends by the accumulated number of variables for the 1- and 3-year mortality prediction models. The 1- and 3-year accuracy trends of mortality prediction model for RF indicated that only the top 14 and 12 important variables were required to achieve the maximum accuracy, respectively.

Table 3 summarizes the rankings of variables for CHD mortality risk via all of the five ML methods. The top half of Table 3 displays the average ranking of variables from DT, RF, GB, and XGB before reaching the maximum accuracy for the 1- and 3-year models. For the 1-year model, the top 14 variables included age, post-HD creatinine, AST, total bilirubin, post-HD BUN, pre-HD creatinine, Kt/V, CTR, LDL, albumin, iPTH, alkaline phosphatase, aluminum, and TIBC. For the 3-year model, the top 12 variables included age, AST, CTR, pre-HD creatinine, alkaline phosphatase, AC blood glucose, iPTH, iCa, post-HD creatinine, ferritin, ALT, and hematocrit. At the bottom half of Table 3, we list the significant variables identified by the LGR model before being used in the modified model. The significant variables in the 1-year model were age, CTR, ferritin, and ALT, whereas those in the 3-year model were age, CTR, iPTH, sex, alkaline phosphatase, pre-HD creatinine, post-HD creatinine, and phosphorus.

**Table 3:** The rankings of variables for CHD mortality risk via different machine learning models.

Rank	The average ranking from Decision Tree (DT), Random Forest (RF), Gradient boosting (GB) and eXtreme Gradient Boosting (XGB)	
	1-year mortality	3-year mortality
1	Age	Age
2	Creatinine (post-HD)	AST
3	AST	Cardiothoracic ratio
4	Total bilirubin	Creatinine (pre-HD)
5	BUN (post-HD)	Alkaline phosphatase
6	Creatinine (pre-HD)	AC blood sugar
7	Kt/V	iPTH
8	Cardiothoracic ratio	iCa
9	LDL	Creatinine (post-HD)
10	Albumin	Ferritin
11	iPTH	ALT
12	Alkaline phosphatase	Hematocrit
13	Aluminum	
14	TIBC	

Rank	Logistic Regression (LGR)	
	1-year mortality	3-year mortality
1	Age	Age
2	Cardiothoracic ratio	Cardiothoracic ratio
3	Ferritin	iPTH
4	ALT	Sex
5		Alkaline phosphatase
6		Creatinine (pre-HD)
7		Creatinine (post-HD)
8		Phosphate (pre-HD)

*CHD* chronic hemodialysis, *Kt/V* urea kinetic, *BUN* blood urea nitrogen, *iPTH* intact parathyroid hormone, *AST* aspartate aminotransferase, *ALT* alanine transaminase, *LDL* low-density lipoprotein, *TIBC* total iron binding capacity.

### Survival prediction performance between the RF with stepwise remodeling and the rebuilt LGR

We used the highest average rank variables from DT, RF, GB, and XGB for the RF with stepwise remodeling method. The results were then compared with those of the rebuilt LGR model. Table 4 compares the survival prediction performance between RF stepwise modeling and rebuilt LGR in terms of accuracy, recall, specificity, and AUC. In the 1-year prediction model, the accuracy of RF stepwise modeling was 0.940, whereas that of the rebuilt LGR was 0.937. The specificity was 1.000 in the RF and 0.996 in the LGR, with AUCs of 0.727 and 0.576, respectively. In the 3-year prediction model, the accuracy of RF stepwise modeling was 0.801, whereas that of the rebuilt LGR was 0.767. Regarding specificity, RF and LGR obtained 0.989 and 0.940, with AUCs of 0.805 and 0.806, respectively. Overall, the stepwise RF model demonstrated superior predictive performance compared to the traditional LGR method in predicting the mortality of CHD patients.

**Table 4:** The comparison of survival predicts from RF stepwise modeling and rebuild LGR.

Method	Number of variables	Accuracy	Sensitivity	Specificity	AUC
1-year mortality prediction					
RF stepwise modeling	**14**	**0.940**	**0.000**	**1.000**	**0.727**
RF (all variables) (Validation dataset)	44	0.948	0.020	1.000	0.734
RF (all variables) (Testing dataset)	44	0.941	0.000	1.000	0.806
Rebuild LGR	**4**	**0.937**	**0.000**	**0.996**	**0.576**
LGR (All variables) (Validation dataset)	44	0.930	0.161	0.971	0.648
LGR (all variables) (Testing dataset)	44	0.912	0.044	0.967	0.734
3-year mortality prediction					
RF stepwise modeling	**12**	**0.801**	**0.096**	**0.989**	**0.805**
RF (all variables) (Validation dataset)	44	0.794	0.075	0.991	0.751
RF (All variables) (Testing dataset)	44	0.804	0.096	0.992	0.763
Rebuild LGR	**8**	**0.767**	**0.118**	**0.940**	**0.806**
LGR (All variables) (Validation dataset)	44	0.779	0.343	0.899	0.746
LGR (All variables) (testing dataset)	44	0.774	0.243	0.913	0.756

*RF* random forest, *LGR* logistic regression, *AUC* area under curve.

### Advantage of DT algorithm

**Figure 4:**
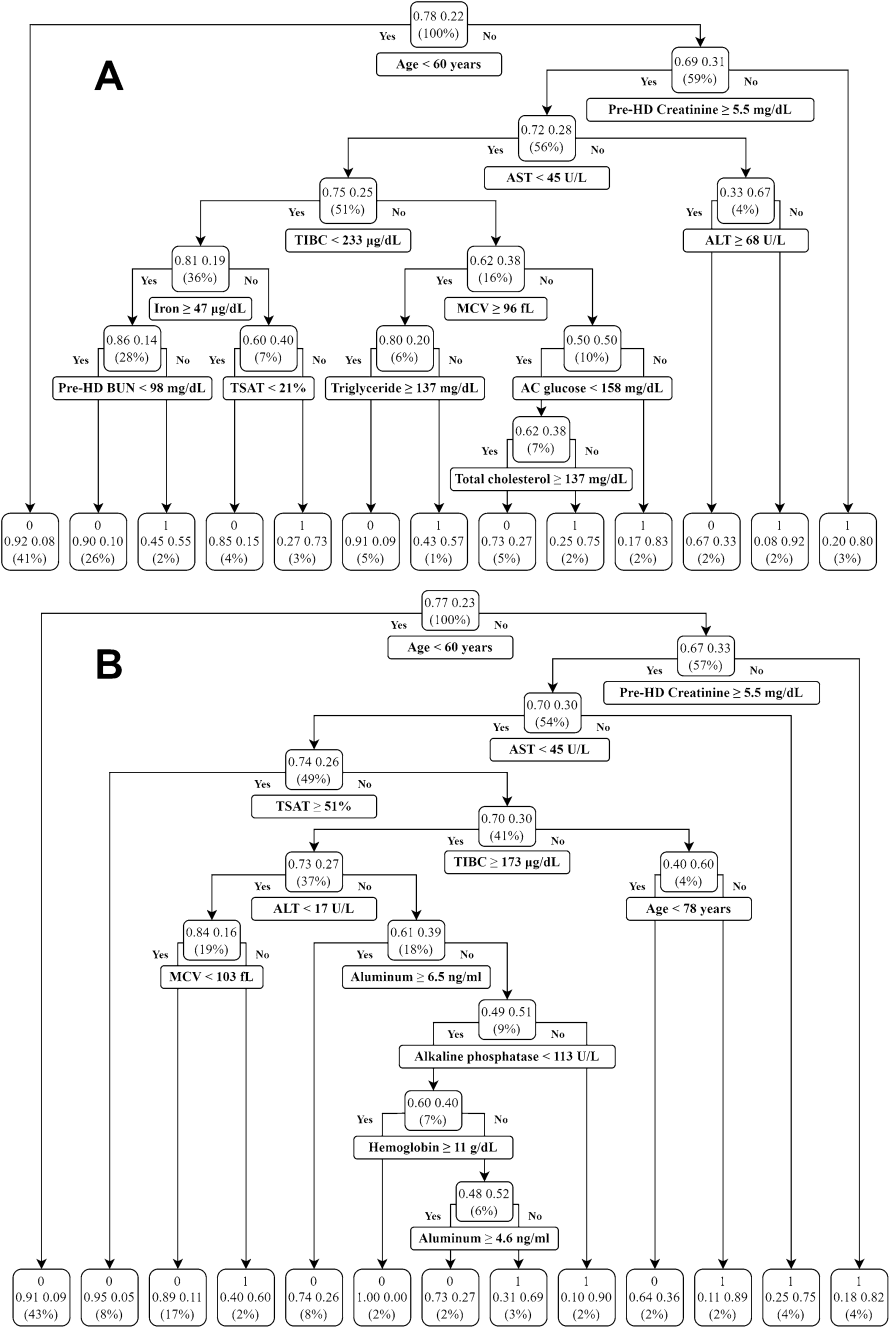
The algorithm of decision tree (DT) in the (**A**) 1-year prediction model (the 7th fold) and (**B**) 3-year prediction model (the 1st fold). *BUN* blood urea nitrogen, *AC* ante Cibum (before meals), *AST* aspartate aminotransferase, *ALT* alanine transaminase, *MCV* mean corpuscular volume, *TIBC* total iron binding capacity, *TSAT* transferrin Saturation.

DT provided a useful and understandable algorithm. Figure 4A shows the algorithm of DT in the 1-year prediction model. The first cutoff criterion was age below 60 years, which accounted for 41% of the validation dataset. The DT model judged the “age below 60 years” as survival (present as “0”), while the actual survival rate was 92%. The rest of the 59% then moved into the second cutoff criterion, that is, pre-HD creatinine level being greater or equal to 5.5 mg/dL; only 3% of the patients failed to meet the criterion. The DT model judged “age greater or equal to 60 years” and “pre-HD creatinine level less than 5.5 mg/dL” as a mortality result (present as “1”), but the actual survival rate was only 20% in the validation dataset. Further cutoff criteria included AST, TIBC, ALT, iron, MCV, pre-HD BUN, TSAT, triglyceride, AC glucose, and total cholesterol level sequentially. Finally, 13 groups had been categorized and moved into the terminal (leaf) of the DT. Figure 4B shows the algorithm of DT in the 3-year prediction model. The first three cutoff criteria were the same (age below 60 years, pre-HD creatinine level greater or equal to 5.5 mg/dL, and AST below 45 U/L). Further cutoff criteria were TSAT, TIBC, ALT, age below 78 years, MCV, aluminum, alkaline phosphatase, and hemoglobin. Likewise, 13 groups had been categorized and moved into the terminal (leaf) of the DT.

## Discussion

**Figure 5:**
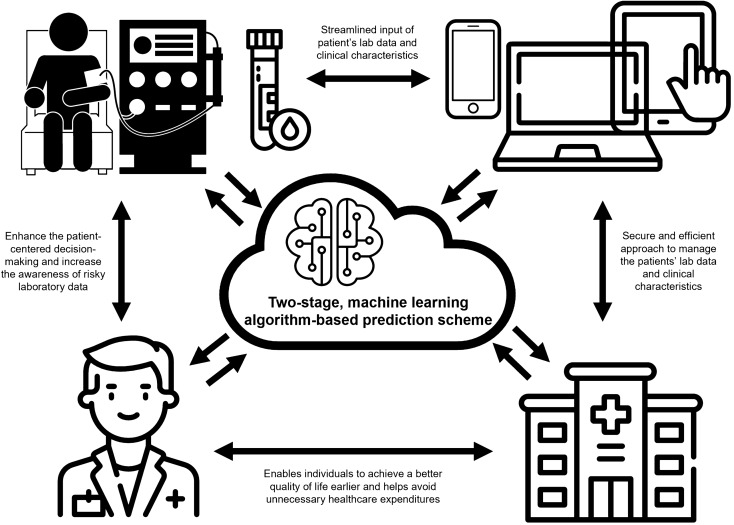
The application of the data-driven machine-learning methods in clinical practice from our study.

Our study adopted five ML methods to manage the best prediction model from different years of mortality in patients with CHD, then successfully developed a two-stage ML algorithm-based prediction scheme to achieve the stepwise RF model, which incorporates the most important factors of CHD mortality risk based on the average rank from DT, RF, GB, and XGB. The stepwise RF model in our study demonstrated superior predictive performance compared to the traditional LGR method for mortality in CHD patients over both 1-year and 3-year model. Finally, we can integrate our proposed ML scheme into the electronic reporting system to enhance patient care (Fig. 5).

All five ML models individually provided solid and consistent performance in predicting the morality of patients with CHD, which were divided into the validation and testing datasets. Regarding the validation and testing datasets from 1- and 3-year mortality prediction model, the RF had better accuracy and area-under-curve results among the five different ML methods. Moreover, the RF only required the top 14 important variables from 1-year accuracy trend and only top 12 variables from the 3-year accuracy trend to reach the maximum accuracy. Interestingly, both the RF stepwise modeling and rebuilt LGR methods needed considerably fewer variables to provide a similar performance from all 44 variables of the 1-year and 3-year models while demonstrating high efficiency in analyzing mortality risk prediction in patients with CHD. Notably, although the DT method did not exhibit the highest accuracy and AUC, it showed a good potential because it provided an algorithm that is very comprehensible and offered some selectable and adjustable variables according to the current clinical trend or physician’s clinical preference.

The top important variables of CHD identified by the five different ML methods provide consistent results. The average rank from all ML methods concluded that age, creatinine (pre- and post-HD), and CTR were the most frequent top variables in all four cutoff periods. Albumin, aluminum, alkaline phosphatase, AST, and AC blood glucose were also top indicators for mortality prediction. Some of these important variables identified by ML methods agree with previous studies. For example, multiple prospective cohort studies reported that CTR^25–27^, elevated serum alkaline phosphatase^28–31^, and lower serum albumin levels^32–34^ are associated with higher mortality risk in patients with CHD. Higher serum aluminum levels also represent as a mortality risk factor^35–37^ and even has some potential associations with CTR, although the cause of the relationship or mechanism is still vague^38^.

Serum creatinine levels before and after HD were highlighted as the top-ranked variables from the ML models and also frequently measured in clinical practice. Walther et al. also concluded that pre-dialysis and interdialytic change of serum creatinine is highly related to mortality in patients undergoing HD^39^. However, muscle lean mass^40–43^, infection status^44,45^, severe illness^46,47^ and poor nutrition status^39,45^ that affects the metabolism and catabolism may influence the serum creatinine level. Some studies investigated the modified creatinine index (mCI) for better mortality prediction^40,43,44,48^. However, the baseline characteristic of serum creatinine level in patients with CHD could be more varied in different study periods, making it very difficult to define in clinical practice. Moreover, the mCI incorporated age, sex, and Kt/V (urea kinetics) into the formula, resulting in multifactorial interference and potentially misleading the prediction bias.

URR, one of the most common indicators of HD dose delivery, is generally associated with decreased mortality^49–52^. Unexpectedly, the URR in our study did not match the top variable factors, probably because the URR in the survival group (1-year subgroup: 0.73 ± 0.06; 3-year subgroup: 0.73 ± 0.06) and mortality group (0.76 ± 0.07 and 0.74 ± 0.06 respectively) were both in optimized dose (≥ 0.65) without statistically significance. Moreover, URR is higher in patients with sarcopenia or malnutrition, leading to higher mortality risk in those with CHD. McClellan et al. concluded that a URR of 0.70–0.74 has an increasing trend of mortality risk compared with 0.65–0.69^53^. Considering that the URR is also affected by urea distribution volume changes and urea generation during HD, the 2015 National Kidney Foundation’s Kidney Disease Outcomes Quality Initiative (KDOQI) recommends the targeted single pool Kt/V (spKt/V) for the dialysis adequacy instead of URR^52^.

Table 5 presents a range of data-driven ML methods employed for identifying risk factors and modifying mortality prediction in patients with CHD. Victoria Garcia-Montemayor et al. concluded that RF is adequate for mortality prediction in patients with CHD, superior to LGR^20^. According to Kaixiang Sheng et al., XGB can effectively identify high-risk patients within 1 year after HD initiation^19^. In the study of Covadonga Díez-Sanmartín et al., combining XGBoost method with the corresponding Kaplan–Meier curve presentation to evaluate the risk profile of patients undergoing dialysis demonstrated very high accuracy, specificity, and AUC results^16^. Oguz Akbilgic et al. also used RF to identify the risk factors of mortality within 1 year after dialysis introduction and inferred that RF also had a strong prediction performance compared with other ML methods, such as artificial neural networks, support vector machines, and k-nearest neighbors algorithm^21^. Furthermore, Cheng-Hong Yang et al. revealed that whale optimization algorithm with full-adjusted-Cox proportional hazards (WOA-CoxPH) could evaluate risks better than RF and typical Cox proportional hazards (CoxPH) in patients with CHD^54^. However, these previous studies had a relatively short prediction period, mostly within 2 years. Our study emphasizes the characteristics of patients with CHD and has effectively achieved a robust prediction performance for up to 3 years using the stepwise RF model, modified from two-stage ML algorithm-based prediction scheme. Figure 5 demonstrate our approach of the AI system by incorporating the top numerical risk variables selected by various ML methods, our study model can effectively assist physicians, caregivers, and patients in predicting short-term post-dialysis mortality outcomes. This model allows for enhanced patient-centered decision-making and increased awareness about laboratory data that could be risk factors, especially for patients with a high short-term mortality risk. It also enables individuals to achieve a better quality of life earlier and helps avoid unnecessary healthcare expenditures.

**Table 5:** Lecture review of the mortality prediction model in chronic hemodialysis patients.

Study	Country	Numbers	Ages (years)	Male (%)	Dialysis type	Follow-up times (Years)	Reporting dataset	Algorithms	Optimal model	AUC	Conclusion
Oguz Akbilgic et al. (2019)	U.S	27,615	68.7 ± 11.2	98.1	HD	1.0	Training and Testing	RF, ANN, KNN	RF	0.7185 (30-day) 0.7446 (90-day) 0.7504 (180-day) 0.7488 (365-day)	RF, ANN and KNN demonstrated robust prediction performance in identifying mortality risk factors within the first year of dialysis initiation
Victoria Garcia-Montemayor et al. (2020)	Spain	1,571	62.33 ± 15.89	61	HD	2.0	Training and Testing	LGR and RF	RF	0.7175 (6-month) 0.7331 (1-year) 0.7259 (2-year)	RF outperforms LGR in developing mortality prediction models for HD patients
Kaixiang Sheng et al. (2020)	China	5,351^(1)^ 5,828^(2)^	51.67 ± 16.48^(3)^ 62.53 ± 16.20^(4)^ 52.61 ± 16.59^(5)^ 62.53 ± 16.20^(6)^	61.58^(7)^ 60.47^(8)^ 62.01^(9)^ 60.71^(10)^	HD	1.0	Training and Testing	XGB	XGB	0.83^(11)^ 0.85^(12)^	XGB effectively identify the high-risk patients within one year after the HD initiation
Cheng-Hong Yang et al. (2022)	Taiwan	829	≥ 65 years: 35.22%^(13)^	45.36	HD	5.0	Training and Testing	CoxPH Stepwise- CoxPH WOA- CoxPH RSF-CoxPH Kaplan–Meier	WOA- CoxPH	0.7404 (CoxPH) 0.7388 (Stepwise-CoxPH) 0.7406 (RSF-CoxPH) 0.7409 (WOA- CoxPH)	WOA-CoxPH demonstrated superior risk assessment performance in HD patients compared to RSF-CoxPH and typical selection CoxPH models
Covadonga Díez-Sanmartín et al. (2023)	U.S.^(14)^	44,663	≥ 60 years: 45.83%^(15)^	62.09^(16)^	HD	6.07^(17)^	Training	XGB, Kaplan–Meier	XGB, Kaplan–Meier	99.08 (Multi-class AUC with 8 clusters)	XGB combined with K–M demonstrates exceptional accuracy, specificity, and area under curve (AUC) outcomes
Our study	Taiwan	800	63.30 ± 13.26	50.63	HD	3.0	Validation (Training) and Testing	LGR, DT, RF, GB, XGB	Stepwise RF	0.727 (1-year) 0.805 (3-year)	Stepwise RF provided superior performance in predicting 1-year and 3-year mortality risks from CHD patients

*HD* hemodialysis, *PD* peritoneal dialysis, *PRISMA* preferred reporting items for systematic reviews and meta-analyses, *MOOSE* meta-analyses of observational studies in epidemiology, *NT-proBNP* N-terminal pro-brain natriuretic peptide, *suPAR* soluble urokinase plasminogen activator receptor, *CRP* C-reactive protein, *LGR* logistic regression, *DT* decision tree, *RF* random forest, *GB* gradient boosting, *XGB* eXtreme gradient boosting, *ANN* artificial neural networks, *KNN* k-nearest neighbors algorithm, *CoxPH* full-adjusted-Cox proportional hazards.

* The study by Kaixiang Sheng et al. involves 5,351 training cohort from a single center (1) and 5,828 testing cohort cases from 97 renal centers (2). Data obtained at dialysis initiation (3, 4, 7, 8, 11) and data 0–3 months after dialysis initiation (5, 6, 9, 10, 12). The average age and male ratio are also shown in the training dataset (3, 5, 7, 9) and testing data set (4, 6, 8, 10), respectively.

** The study by Cheng-Hong Yang et al. did not mention the overall study population average age, only provide the age ≥ 65 years group percentage instead (13).

*** The study by Covadonga Díez-Sanmartín et al. utilized the dataset from the Organ Procurement and Transplantation Network (OPTN), consisting of medical data from patients located within the United States (14). The average age (15) and male ratio (16) of the study population is weighted average from all 8 clusters. The follow-up time is the highest survival mean time (17) from the study (Cluster 2).

This study has some limitations. First, the top variables modified by ML only provide the relationship of mortality in patients with CHD but not infer the positive or negative associations from these variables. Moreover, not all the top variables agree with previous study results, especially serum creatinine level, which can be affected by various clinical conditions that may mislead the data-driven ML results. In the future implementation of ML-identified variables to an unknown disease, these variables should be clinically investigated further. Second, we initially extended the analysis cutoff period up to 7 years but then only selected within 3 years to avoid data-censoring risk of bias by a high mortality rate after dialysis initiation. A long-term prediction model may need larger data and even longer study period for better qualification and quantification. Third, our dataset only contained a composite parameter for CVD. However, prognoses may vary among different CVD categories in CHD patients. Additional research may be required to examine the effects of subclassifications of CVD on mortality. Fourth, the individuals in our study were chosen from a pool that did not encompass recently hospitalized patients dealing with cardiovascular or infectious concerns. This subset was anticipated to exhibit a greater likelihood of survival compared to those who had recently been hospitalized, a factor that could potentially skew our study results. Therefore, our model may only be applicable to the CHD patients who are relatively stable. Finally, the developed prediction models by ML methods in our study are limited to a single medical center. Hence, the model modified from our study population may not be applicable to other similar at-risk research groups. Future studies on ML such as federated learning that incorporates multiple medical centers and research groups could be helpful for improving predictive performance and strengthening clinical decisions.

## Conclusion

The adoption of the stepwise RF model, modified from two-stage ML algorithm-based prediction scheme, enhances patient-centered decision-making, and improves outcomes, particularly for patients with a high short-term mortality risk in both 1-year and 3-year periods. The findings of this study can offer valuable information to nephrologists, increasing awareness about risky laboratory data. However, for longer prediction periods, future studies should consider incorporating larger study populations and diverse groups to further enhance predictive performance.

## Data Availability

The supporting data can only be accessed by legitimate researchers under a non-disclosure agreement because of confidentiality obligations. Further information on the data and instructions for requesting access can be obtained from the corresponding author.
